# The impact of the March 2020 lockdown on the cardiometabolic risk factors of male forensic and rehabilitation patients

**DOI:** 10.1192/bjo.2021.658

**Published:** 2021-06-18

**Authors:** Nicholas Dodough, Jaspreet Phull, Jaswant Singh, Jackie Sendell

**Affiliations:** Lincolnshire Partnership Foundation Trust

## Abstract

**Aims:**

To explore the impact of the March 2020 lockdown restrictions on the cardiometabolic risk factors of male forensic and rehabilitation inpatients in one NHS trust in the United Kingdom.

**Method:**

Retrospective data from September 2019 to September 2020 (six months before and after the 23 March 2020 lockdown) was collected by evaluating the health records of male patients in a low secure forensic ward and two rehabilitation units.

**Result:**

The number of patients with BMI values within the study period was 34 while the number of patients with blood results was 26. This study showed that the average BMI six months before the start of the March lockdown was 29.8 kg/m2 while the average BMI at the end of six months after the lockdown was 31.8 kg/m2.

The results from the 6-month interval before the March 2020 lockdown (M = 0, SD = 0) and the 6- month interval after the March 2020 lockdown (M = 0.9, SD = 4.16) indicate that the March 2020 lockdown resulted in an increase of BMI, t (5) = 2.42, P = 0.036. The result is significant at p < 0.05

8.8% of patients had an increase in their doses of antihypertensive agents after the lockdown whereas no patients had an increase of dose before the lockdown. 7.7% of patients had an HBA1c of more than 48 mmol/L after the lockdown compared to 3.8% before the lockdown. The serum triglycerides and total cholesterol levels also increased after the lockdown with an average increase of 0.17 mmol/L and 0.25 mmol/L respectively. The average serum HDL levels decreased after the lockdown with an average decrease of 0.06 mmol/L.

**Conclusion:**

There appears to be a positive correlation between the onset of the March 2020 lockdown and an increase of BMI, worsening of blood pressure, glycemic control and lipid metabolism.

Limitations

1. Waist circumference was not measured during the study period preventing us from examining all of the features of metabolic syndrome.
2. This study did not look at the levels of physical activity (such as access to section 17 leave) and dietary habits before and after the March 2020 lockdown which may explain the results found.

Recommendations

To raise metabolic awareness of the impact of the lockdown restrictions on cardiometabolic risk in people with SMI and the general public.

